# The association between sleep quality and cognitive impairment among a multi-ethnic population of middle-aged and older adults in Western China: a multi-center cross-sectional study

**DOI:** 10.3389/fpubh.2025.1500027

**Published:** 2025-04-17

**Authors:** Yuexia Hu, Xin Xia, Huixian Li, Yuqing Xie, Xin Tian, Yun Li, Jirong Yue, Birong Dong, Yanyan Wang

**Affiliations:** ^1^Healthcare Innovation Research Laboratory, West China School of Nursing & National Clinical Research Center for Geriatrics, Science and Technology Department, West China Hospital, Sichuan University, Chengdu, China; ^2^National Clinical Research Center for Geriatrics, West China Hospital, Sichuan University, Chengdu, Sichuan, China

**Keywords:** cognitive impairment, sleep quality, daytime dysfunction, Alzheimer’s disease, multi-ethnic

## Abstract

**Objective:**

This study aims to investigate the relationship between sleep quality and cognitive impairment in middle-aged and older adults living in Western China.

**Methods:**

Baseline data from the West China Health and Aging Trend (WCHAT) study were utilized, with enrollment of participants aged 50 years or older. Sleep quality was assessed using the Pittsburgh Sleep Quality Index (PSQI), where a PSQI score > 5 indicated poor sleep quality. The cognitive status was evaluated using the 10-item Short Portable Mental Status Questionnaire (SPMSQ). Multinomial logistic regression was applied to estimate odds ratios (ORs) and 95% confidence intervals (CIs).

**Results:**

A total of 6,728 middle-aged and older adults dwelling in western China (age = 62.39 ± 8.925 years, 2,520 males and 4,208 females) were included in the analysis. The prevalence of mild cognitive impairment was 11.0%, with 4.3% having moderate to severe cognitive impairment. Poor sleep quality was found in 47.7% of participants. After adjusting for age, gender, education, marital status, and chronic disease, poor sleep quality (OR = 1.29, 95% CI: 1.1–1.52, *p* = 0.002) was associated with a higher risk of mild cognitive impairment. Among specific sleep dimensions, there is a significant association between high daytime dysfunction and mild cognitive impairment (adjusted OR = 1.96, 95% CI: 1.45–2.62) as well as moderate to severe cognitive impairment (adjusted OR = 3.15, 95% CI: 2.09–4.73) in the middle-aged and older adults residing in multi-ethnic areas of western China. Besides, a sleep duration of 6–7 h was associated with a reduced risk of mild cognitive impairment (OR = 0.80, 95% CI: 0.65–0.98) and moderate to severe cognitive impairment (OR = 0.71, 95% CI: 0.51–0.99). Stratified analysis showed that poor sleep quality—particularly daytime dysfunction—was significantly associated with moderate to severe cognitive impairment in Han (OR = 4.14, 95%CI: 1.65–10.23), Tibetan (OR = 3.45, 95% CI: 1.42–8.1), and Yi (OR = 4.04, 95%CI: 1.46–10.97) participants, but not in Uyghur and Qiang groups.

**Conclusion:**

Sleep quality is significantly associated with cognitive impairment among middle-aged and older adults in Western China, particularly concerning the components of daytime dysfunction.

## 1 Introduction

The rising global aging population has led to an increase in cognitive impairment among the older adults, posing a significant public health challenge worldwide (1–3). Cognitive impairment refers to a group of symptoms that affect thinking, memory, language, attention, perception, and executive functions and are most common in the older adults (4). Among adults aged 65 and above, the incidence of cognitive impairment is between 10 and 20% (5), and the incidence doubles every 5 years (6). Alzheimer’s Disease (AD) is one of the leading causes of cognitive impairment, accounting for approximately 60–80% of all dementia cases (7, 8), affecting millions of people worldwide. In China, the annual total costs of AD are predicted to reach US $507.49 billion in 2030 and US $1.89 trillion in 2050 and will confer a heavy economic burden on society and families (9). Given the current absence of definitive cures and effective drugs for Alzheimer’s disease (AD), targeted early identification and intervention of modifiable risk factors can effectively delay the progression of cognitive impairment and subsequent Alzheimer’s disease (10, 11), especially in regions with relatively underdeveloped medical infrastructure.

Sleep plays a pivotal role in maintaining circadian rhythms, homeostasis, and neurohormonal process regulation (12), which are essential for preserving physical and cognitive functions (13). Poor sleep quality has also been associated with cognitive decline and Aβ deposition (14), contributing to Alzheimer’s Disease (AD) pathology (15). People with poor sleep performance worse in individual episodic memory (16), working memory (17), executive function, and language (18). Amount evidence shows that self-reported sleep quality can be associated with cognition in older adults (19–21). Interestingly, race/ethnicity modified these associations: compared to estimated effects among White participants, poorer global sleep quality was associated with larger effects on decline in executive function among Black participants, and estimated effects of some individual sleep quality components were also modified by race or ethnicity (22). However, few studies have investigated the association between sleep and cognition among different ethnic groups in China.

Western China is the area with the highest concentration of ethnic minorities in China. About 70% of ethnic minorities (44 ethnic minorities) live in western China. The proportion of non-Han ethnic groups among the people in western China is about 33% (23). Compared to the eastern coastal regions, the western regions of China face relatively insufficient medical resources and a significant urban–rural disparity (24, 25). Consequently, limited research has examined the association between cognitive impairment and sleep quality among middle-aged and older adults in western China, especially in remote, rural, and ethnic minority communities. Understanding this association could provide valuable insights into the cognitive impact of sleep and guide interventions to delay the progression of AD.

To the best of our knowledge, this is a large-scale study to explore the relationship between sleep quality and cognitive impairment in a multi-ethnic population of middle-aged and older adults in Western China, a region with significant ethnic diversity and limited healthcare resources. Unlike previous studies that primarily focused on urban populations or Eastern China, our research investigates this association in a rural, under-researched area characterized by diverse cultural backgrounds and healthcare disparities. This research might uniquely contributes to the understanding of how sleep quality affects cognitive health in a population with diverse cultural backgrounds and limited access to healthcare services.

## 2 Materials and methods

### 2.1 Data collection and study population

Data were derived from the West-China health and aging trend study (WCHAT) collected from July to December in 2018, a longitudinal multi-center cohort research conducted in western China to assess the health and aging status in the multi-ethnic region. Data were collected from 4 provinces including Yunnan, Guizhou, Sichuan, and Xinjiang. All participants aged 50 years or older were enrolled. Participants were recruited by convenience and asked verbally by the researchers about their willingness to take part in the study. Before the investigation, informed consent was signed and obtained by each participant. Initially, we recruited 7,536 community-dwelling multi-ethnic Chinese in total. The cohort study began in 2018 and was approved by the Ethics Committee of West China Hospital, Sichuan University (reference: 2017–445) and was conducted in accordance with the 2013 version of the Declaration of Helsinki. The study was registered at the Chinese Clinical Trial Registry under number ChiCTR1800018895. Trained interviewers collected the baseline data through face-to-face interviews and physical examination. A written informed consent was obtained from all participants (or their legal proxies for those who were unable to sign their names).

### 2.2 Sleep quality measures

Sleep quality was assessed using the Pittsburgh Sleep Quality Index (PSQI). The questionnaire included seven components (subjective sleep quality, sleep latency, sleep duration, habitual sleep efficiency, sleep disturbances, use of sleep medications and daytime dysfunction), and each component scores range from 0 to 3 and a global score ranging from 0 to 21, with higher scores indicating worse sleep quality. Poor global sleep quality was defined by a global PSQI >5 (26).

### 2.3 Cognitive impairment measures

Cognitive status was measured using the 10-item Short Portable Mental Status Questionnaire (SPMSQ). Scores of 0–2 indicated complete cognitive function, 3–4 indicated mild cognitive impairment, and 5–10 indicated moderate to severe cognitive impairment, and this assessment should be based on the education level (27).

### 2.4 Covariates

Information regarding factors that can be involved in both sleep quality and cognition function was collected through face-to-face interviews: age, gender, educational level (no formal education, elementary school, middle school, high school and high above), marital status (with/without spouse), chronic diseases (with/without). We defined smoking history and drinking alcohol history as a period of regular use of smoking or drinking (yes/no). Body Mass Index (BMI) was categorized based on established criteria as follows: underweight (BMI < 18.5 kg/m^2^), normal weight (18.5 ≤ BMI < 24.0 kg/m^2^), overweight (24.0 ≤ BMI < 28.0 kg/m^2^), and obesity (BMI ≥ 28.0 kg/m^2^) (28).

### 2.5 Statistical analysis

One-sample Kolmogorov–Smirnov test was used to determine the normalized distribution of variables. The data were presented as medians (interquartile ranges) or means (standard deviation) or frequencies. The Pearson chi-squared test was used for categorical variables and Kruskal–Wallis H test or one-way analysis of variance (ANOVA) for continuous variables. The association between sleep quality and cognitive impairment was analyzed using a single multinomial logistic regression model, adjusted for age, gender, educational level, marital status, and chronic disease. To examine differences across ethnic groups, we conducted stratified analyses by ethnicity and applied the same regression method within each subgroup. All statistical analyses were performed using R (version 4.1.3). A two-tailed *p*-value of <0.05 was considered statistically significant.

## 3 Results

**Figure 1:**
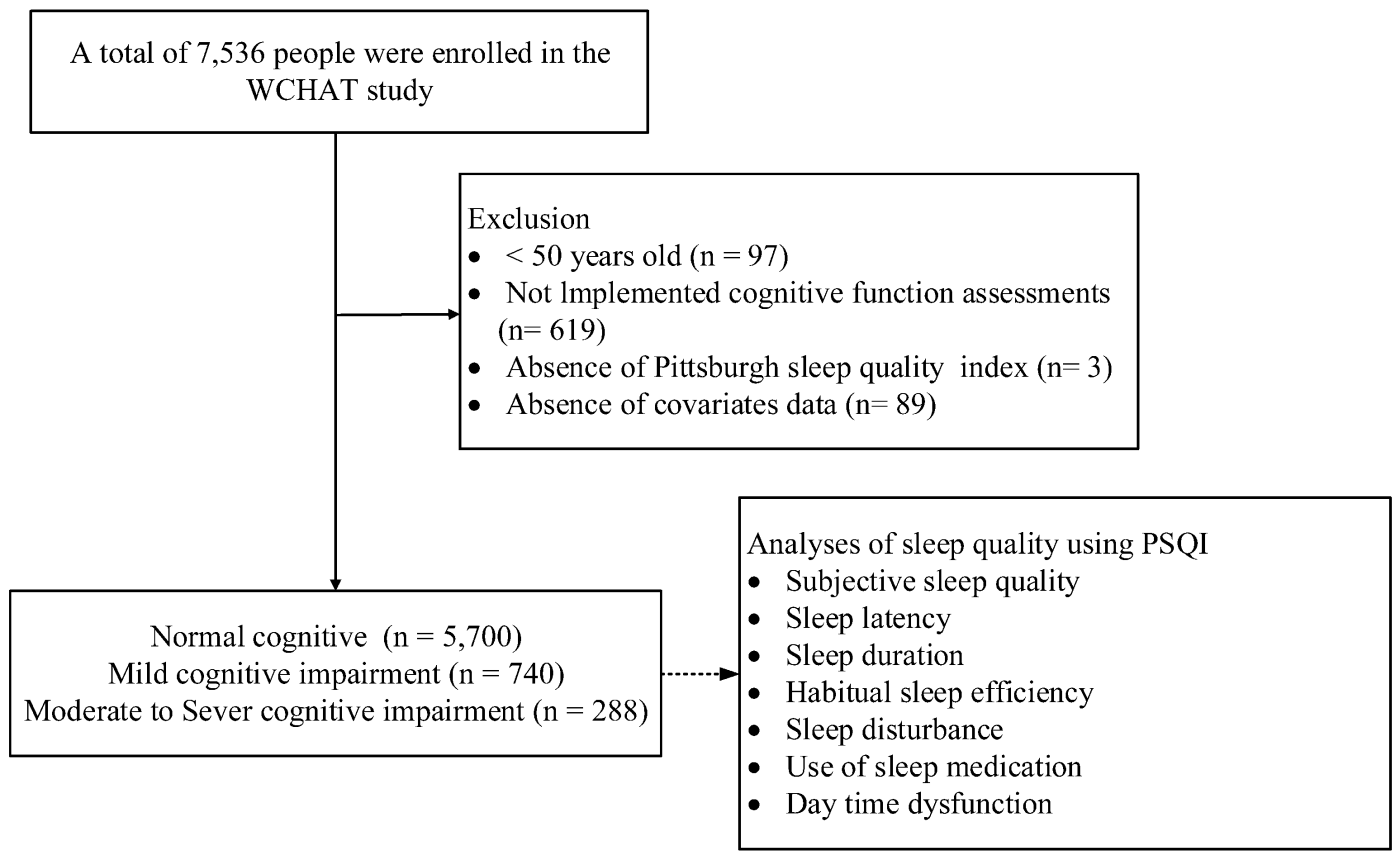
A schematic illustration of the participant selection process used in this study. Of the total 7,536 participants, 740 with mild cognitive impairment, 288 participants with moderate to severe cognitive impairment and 5,700 control participants (normal cognition) were included in the analysis.

Among the 6,728 participants (mean [SD] age: 62.39 [8.25] years; 62.5% female, 37.5% male) aged 50 years or older (range: 50–95 years), 740 (11.0%) were found to have mild cognitive impairment, while 288 (4.3%) had moderate to severe cognitive impairment (Figure 1). Additionally, 3,206 (47.7%) participants were identified as having poor sleep quality.

Table 1 illustrates the characteristics of participants based on their cognitive impairments. Significant differences were observed among participants with normal cognition, mild cognitive impairment, and moderate to severe cognitive impairment regarding, age, gender, ethnics, sleep quality, educational level, marital status, smoking history, drinking alcohol and BMI. Compared to participants with normal cognition, individuals with mild or moderate to severe cognitive impairment were more likely to be older, female, individuals with lower BMI, higher PSQI score, lower educational levels, without a spouse, non-smokers, and non-drinkers (Table 1).

**Table 1:** Characteristics of the participants according to cognitive impairment status.

Characters	Total	Normal	Mild CI	Mod/sev CI	*p*-value
Total Number, *n*	6,728	5,700	740	288	
Age (mean (SD))	62.39 (8.3)	61.95 (8.0)	64.12 (8.7)	66.55 (9.3)	<0.001^a^
Gender (%)					<0.001^b^
Male	2,520 (37.5)	2,289 (40.2)	180 (24.3)	51 (17.7)	
Female	4,208 (62.5)	3,411 (59.8)	560 (75.7)	237 (82.3)	
Ethic (%)					<0.001^b^
Han	2,451 (36.4)	2,222 (39.0)	178 (24.1)	51 (17.7)	
Tibetan	1,296 (19.3)	1,058 (18.6)	169 (22.8)	69 (24.0)	
Qiang	1,274 (18.9)	1,079 (18.9)	154 (20.8)	41 (14.2)	
Yi	612 (9.1)	435 (7.6)	105 (14.2)	72 (25.0)	
Uyghur	572 (8.5)	489 (8.6)	70 (9.5)	13 (4.5)	
Others	523 (7.8)	417 (7.3)	64 (8.6)	42 (14.6)	
PSQI (mean (SD))	5.99 (3.5)	5.87 (3.5)	6.58 (3.5)	6.83 (3.9)	<0.001^a^
Global sleep quality					<0.001^b^
Good (PSQI≤5)	3,522 (52.3)	3,080 (54.0)	320 (43.2)	122 (42.4)	
Poor (PSQI>5)	3,206 (47.7)	2,620 (46.0)	420 (56.8)	166 (57.6)	
Subjective sleep quality (%)					<0.001^b^
Very good (0 score)	1,227 (18.2)	1,083 (19.0)	112 (15.1)	32 (11.1)	
Fairly good (1 score)	3,546 (52.7)	3,021 (53.0)	383 (51.8)	142 (49.3)	
Fairly bad (2 score)	1,663 (24.7)	1,355 (23.8)	211 (28.5)	97 (33.7)	
Very bad (3 score)	292 (4.3)	241 (4.2)	34 (4.6)	17 (5.9)	
Sleep latency (%)					<0.001^b^
0 (0 score)	2,012 (29.9)	1,764 (30.9)	180 (24.3)	68 (23.6)	
1–2 (1 score)	2,098 (31.2)	1,800 (31.6)	215 (29.1)	83 (28.8)	
3–4 (2 score)	1,470 (21.8)	1,200 (21.1)	191 (25.8)	79 (27.4)	
5–6 (3 score)	1,148 (17.1)	936 (16.4)	154 (20.8)	58 (20.1)	
Sleep duration (%)					0.036^b^
>7 h (0 score)	3,694 (54.9)	3,091 (54.2)	433 (58.5)	170 (59.0)	
6–7 h (1 score)	1,538 (22.9)	1,343 (23.6)	144 (19.5)	51 (17.7)	
5–6 h (2 score)	1,205 (17.9)	1,014 (17.8)	133 (18.0)	58 (20.1)	
<5 h (3 score)	291 (4.3)	252 (4.4)	30 (4.1)	9 (3.1)	
Sleep efficiency (%)					0.883
≥85% (0 score)	4,617 (68.6)	3,920 (68.8)	501 (67.7)	196 (68.1)	
75–84% (1 score)	1,006 (15.0)	846 (14.8)	110 (14.9)	50 (17.4)	
65–74% (2 score)	423 (6.3)	357 (6.3)	50 (6.8)	16 (5.6)	
<65% (3 score)	682 (10.1)	577 (10.1)	79 (10.7)	26 (9.0)	
Sleep disorder (%)					<0.001^b^
0 (0 score)	256 (3.8)	221 (3.9)	23 (3.1)	12 (4.2)	
1–9 (1 score)	4,356 (64.7)	3,766 (66.1)	426 (57.6)	164 (56.9)	
10–18 (2 score)	2,025 (30.1)	1,652 (29.0)	273 (36.9)	100 (34.7)	
19–28 (3 score)	91 (1.4)	61 (1.1)	18 (2.4)	12 (4.2)	
Hypnotic drugs (%)					0.321
Not during the last month (0 score)	6,533 (97.1)	5,533 (97.1)	718 (97.0)	282 (97.9)	
Less than once a week (1 score)	79 (1.2)	69 (1.2)	9 (1.2)	1 (0.3)	
Once or twice a week (2 score)	50 (0.7)	46 (0.8)	4 (0.5)	0 (0.0)	
Three or more times a week (3 score)	66 (1.0)	52 (0.9)	9 (1.2)	5 (1.7)	
Daytime dysfunction (%)					<0.001^b^
Not at all (0 score)	2,779 (41.3)	2,485 (43.6)	218 (29.5)	76 (26.4)	
1–2 (1 score)	2,097 (31.2)	1,781 (31.2)	239 (32.3)	77 (26.7)	
3–4 (2 score)	1,369 (20.3)	1,079 (18.9)	204 (27.6)	86 (29.9)	
5–6 (3 score)	483 (7.2)	355 (6.2)	79 (10.7)	49 (17.0)	
Educational level (%)					<0.001^b^
No formal education	1,859 (27.6)	1,279 (22.4)	380 (51.4)	200 (69.4)	
Elementary school	2,283 (33.9)	2,036 (35.7)	194 (26.2)	53 (18.4)	
Middle school	1,453 (21.6)	1,363 (23.9)	74 (10.0)	16 (5.6)	
High school	413 (6.1)	374 (6.6)	30 (4.1)	9 (3.1)	
High above	720 (10.7)	648 (11.4)	62 (8.4)	10 (3.5)	
Marital status (%)					<0.001^b^
Without spouse	1,108 (16.5)	856 (15.0)	160 (21.6)	92 (31.9)	
Have Spouse	5,620 (83.5)	4,844 (85.0)	580 (78.4)	196 (68.1)	
Smoking history (%)					<0.001^b^
No	5,438 (80.8)	4,544 (79.7)	640 (86.5)	254 (88.2)	
Yes	1,290 (19.2)	1,156 (20.3)	100 (13.5)	34 (11.8)	
Drinking alcohol (%)					<0.001^b^
No	4,993 (74.2)	4,167 (73.1)	587 (79.3)	239 (83.0)	
Yes	1,735 (25.8)	1,533 (26.9)	153 (20.7)	49 (17.0)	
BMIWJ (mean (SD))	25.34 (4.2)	25.42 (4.1)	25.28 (4.7)	24.07 (4.9)	<0.001^a^
BMI (%)					<0.001^b^
Underweight	188 (2.8)	139 (2.4)	30 (4.1)	19 (6.6)	
Normal weight	2,470 (36.7)	2052 (36.0)	278 (37.6)	140 (48.6)	
Overweight	2,512 (37.3)	2,185 (38.3)	242 (32.7)	85 (29.5)	
Obesity	1,558 (23.2)	1,324 (23.2)	190 (25.7)	44 (15.3)	
Chronic diseases (%)					0.335
No	3,680 (54.7)	3,126 (54.8)	389 (52.6)	165 (57.3)	
Yes	3,048 (45.3)	2,574 (45.2)	351 (47.4)	123 (42.7)	

Characteristics of participants based on their cognitive impairment status.

CI, cognitive impairment; Mod/sev, moderate to severe.

^a^Kruskal–Wallis *H* test or one-way analysis of variance (ANOVA) for continuous variables. Significance at *p* < 0.05.

^bPearsons chi-squared test for categorical variables. Significance at p < 0.05.^

After accounting for age, gender, educational level, and marital status, compared to individuals with normal weight, underweight status was associated with an increased risk of mild cognitive impairment (adjusted OR = 1.62, 95% CI = 1.03–2.48, *p* = 0.029), whereas overweight (adjusted OR = 0.64, 95% CI = 0.48–0.86, *p* = 0.003) and obesity (adjusted OR = 0.53, 95% CI = 0.36–0.75, *p* < 0.001) were associated with an elevated risk of moderate-to-severe cognitive impairment. Smoking history and Drinking alcohol was not associated with cognitive impairment. People with poor sleep quality were 1.29 times more likely to have mild cognitive impairment compared to those with good sleep quality (95% CI=: 1.1–1.52, *p* = 0.0019). However, poor sleep quality was not significantly linked to moderate-to-severe cognitive impairment (Table 2).

**Table 2:** Association between ethnicity, sleep quality and cognitive impairment.

Characters	Mild CI OR [95%CI]	*p*-value	Mod/Sev CI OR [95%CI]	*p*-value
Ethic				
Han	1.0 (Ref)		1.0 (Ref)	
Tibetan	2.02 (1.6–2.56)	<0.001^a^	3.26 (2.19–4.90)	<0.001^a^
Qiang	1.56 (1.23–1.98)	<0.001^a^	1.50 (0.96–2.33)	0.075
Yi	3.02 (2.28–3.97)	<0.001^a^	7.62 (5.07–11.53)	<0.001^a^
Uyghur	2.47 (1.78–3.38)	<0.001^a^	2.42 (1.20–4.57)	0.009^a^
Others	2.00 (1.45–2.73)	<0.001^a^	4.84 (3.06–7.63)	<0.001^a^
Global sleep quality				
Good (PSQI ≤ 5)	1.0 (Ref)		1.0 (Ref)	
Poor (PSQI > 5)	1.29 (1.1–1.52)	0.002^a^	1.23 (0.96–1.59)	0.109
Daytime dysfunction				
Not at all (0 score)	1.0 (Ref)		1.0 (Ref)	
1–2 (1 score)	1.41 (1.15–1.72)	<0.001^a^	1.24 (0.88–1.73)	0.214
3–4 (2 score)	1.89 (1.53–2.33)	<0.001^a^	2.15 (1.54–3.01)	<0.001^a^
5–6 (3 score)	1.96 (1.45–2.62)	<0.001^a^	3.15 (2.09–4.73)	<0.001^a^
Subjective sleep quality				
Very good (0 score)	1.0 (Ref)		1.0 (Ref)	
Fairly good (1 score)	1.09 (0.87–1.37)	0.467	1.30 (0.88–1.97)	0.206
Fairly bad (2 score)	1.18 (0.92–1.53)	0.193	1.72 (1.14–2.66)	0.013^a^
Very bad (3 score)	0.91 (0.59–1.38)	0.678	1.34 (0.7–2.51)	0.360
Sleep latency				
0 (0 score)	1.0 (Ref)		1.0 (Ref)	
1–2 (1 score)	1.08 (0.87–1.34)	0.490	0.99 (0.71–1.4)	0.976
3–4 (2 score)	1.36 (1.08–1.7)	0.008^a^	1.37 (0.96–1.94)	0.080
5–6 (3 score)	1.28 (1–1.62)	0.047^a^	1.10 (0.75–1.61)	0.622
Sleep duration				
>7 h (0 score)	1.0 (Ref)		1.0 (Ref)	
6–7 h (1 score)	0.80 (0.65–0.98)	0.030^a^	0.71 (0.51–0.99)	0.046^a^
5–6 h (2 score)	0.90 (0.73–1.11)	0.346	0.94 (0.68–1.29)	0.712
<5 h (3 score)	0.72 (0.47–1.07)	0.116	0.51 (0.24–0.99)	0.066
Habitual sleep efficiency				
>85% (0 score)	1.0 (Ref)		1.0 (Ref)	
75–84% (1 score)	0.95 (0.76–1.19)	0.686	1.07 (0.76–1.49)	0.676
65–74% (2 score)	0.93 (0.67–1.28)	0.681	0.68 (0.38–1.14)	0.169
<65% (3 score)	0.97 (0.74–1.25)	0.812	0.73 (0.46–1.11)	0.163
Sleep disturbance				
Not at all (0 score)	1.0 (Ref)		1.0 (Ref)	
1–9 (1 score)	1.06 (0.69–1.72)	0.792	0.77 (0.42–1.53)	0.421
10–18 (2 score)	1.33 (0.85–2.16)	0.236	0.89 (0.48–1.79)	0.726
19–28 (3 score)	1.92 (0.94–3.88)	0.072	2.27 (0.9–5.75)	0.080
Use of sleep medication				
Not during the past month (0 score)	1.0 (Ref)		1.0 (Ref)	
Less than once a week (1 score)	0.98 (0.45–1.91)	0.956	0.29 (0.02–1.34)	0.219
Once or twice a week (2 score)	0.6 (0.18–1.53)	0.345	0.00 (0–1.44)	0.967
Three or more times a week (3 score)	1.12 (0.5–2.25)	0.761	2 (0.66–4.93)	0.168
BMIWJ				
Normal weight	1.0 (Ref)		1.0 (Ref)	
Underweight	1.62 (1.03–2.48)	0.029^a^	1.68 (0.93–2.9)	0.071
Overweight	0.85 (0.71–1.03)	0.098	0.64 (0.48–0.86)	0.003^a^
Obesity	1.05 (0.85–1.29)	0.636	0.53 (0.36–0.75)	<0.001^a^
Smoking history				
No	1.0 (Ref)		1.0 (Ref)	
Yes	1.08 (0.82–1.42)	0.577	1.14 (0.72–1.77)	0.572
Drinking alcohol				
No	1.0 (Ref)		1.0 (Ref)	
Yes	0.95 (0.77–1.17)	0.645	0.84 (0.59–1.17)	0.302

Adjusted for age, gender, educational level, marital status and chronic disease. Others = other ethnics including Zhuang, Manchu, Hui, Mongolia, Tujia ethnics.

CI, cognitive impairment; Ref, reference.

^aPearsons chi-squared test for categorical variables. Significance at p < 0.05.^

In the analysis of the seven components of the PSQI, individuals with a sleep duration of 6–7 h exhibited a 20% lower likelihood of mild cognitive impairment (adjusted OR = 0.8, 95% confidence interval [CI]: 0.65–0.98) and a 29% lower likelihood of moderate-to-severe cognitive impairment (adjusted OR = 0.71, 95% CI: 0.51–0.99) compared to those with a sleep duration exceeding 7 h. Furthermore, individuals experiencing significant daytime dysfunction were found to have nearly twice the odds of mild cognitive impairment (adjusted OR = 1.96, 95% CI: 1.45–2.62) and more than triple the odds of moderate-to-severe cognitive impairment (adjusted OR = 3.15, 95% CI: 2.09–4.73) compared to those without such dysfunction. Individuals with fairly bad subjective sleep quality exhibited a significantly higher likelihood of developing moderate-to-severe cognitive impairment compared to those with very good subjective sleep quality (adjusted OR = 1.72, 95% CI: 1.14–2.66). A sleep latency score of ≥2 was significantly associated with an increased risk of mild cognitive impairment compared to a sleep latency score of 0 (adjusted OR = 1.72, 95% CI: 1.14–2.66). However, no significant associations were observed between sleep efficiency, sleep disturbances, or the use of sleep medication and cognitive impairment (Table 2).

Cognitive status varied across different ethnic groups. The Tibetan, Yi, Uighur, and other ethnic minorities exhibited a higher prevalence of mild cognitive impairment and moderate-to-severe cognitive impairment compared to the Han population (Tibetan: mild OR = 2.02, 95% CI: 1.60–2.56, moderate-to-severe OR = 3.26, 95% CI: 2.19–4.9; Yi: mild OR = 3.02, 95% CI: 2.28–3.97, moderate-to-severe OR = 7.62, 95% CI: 5.07–11.53; Uighur: mild OR = 2.47, 95% CI: 1.78–3.38, moderate-to-severe OR = 2.42, 95% CI: 1.2–4.57; other ethnic minorities: mild OR = 2.0, 95% CI: 1.45–2.73, moderate-to-severe OR = 4.84, 95% CI: 3.06–7.63). The Qiang ethnic group (OR = 1.56, 95% CI: 1.23–1.98) exhibited an increased risk of mild cognitive impairment (Table 2).

Given the multi-ethnic composition of the study sample and existing evidence of inter-ethnic disparities in sleep and cognition, we conducted stratified analyses by ethnicity to explore potential effect modification. When conducting a stratified analysis of sleep quality and cognitive function across different ethnic groups, it was observed that poor sleep quality was associated with mild cognitive impairment in the Han (OR = 1.55, 95% CI: 1.11–2.16, *p* = 0.0103) and Tibetan (OR = 1.48, 95% CI: 1.05–2.1, *p* = 0.0273). Specifically, high daytime dysfunction, the most sensitive component of PSQI, was significantly correlated with cognitive impairment in the Han (mild OR = 2.71, 95% CI: 1.52–4.72; moderate-to-severe OR = 4.14, 95% CI: 1.65–10.23), Tibetan (mild OR = 2.09, 95% CI: 1.07–3.96; moderate-to-severe OR = 3.45, 95% CI: 1.42–8.1), and Yi (mild OR = 3.28, 95% CI: 1.17–8.75; moderate-to-severe OR = 4.04, 95% CI: 1.46–10.97) groups (*p* < 0.05), but not in the Uyghur and Qiang groups (Table 3). The associations between the remaining six components of the PSQI and cognitive impairment in different ethnic groups are listed in Supplementary Tables S1–S6.

**Table 3:** Associations between daytime dysfunction and cognitive impairment across ethnic groups.

Daytime dysfunction	Mild CI, OR [95%CI]	*p*-value	Mod/Sev CI, OR [95%CI]	*p*-value
Han				
Not at all (0 score)	1.0 (Ref)		1.0 (Ref)	
1–2 (1 score)	1.48 (0.99–2.23)	0.057	1.22 (0.52–2.82)	0.635
3–4 (2 score)	2.47 (1.61–3.78)	<0.001^a^	2.66 (1.22–5.88)	0.014^a^
5–6 (3 score)	2.71 (1.52–4.72)	<0.001^a^	4.14 (1.65–10.23)	0.002^a^
Tibetan				
Not at all (0 score)	1.0 (Ref)		1.0 (Ref)	
1–2 (1 score)	1.32 (0.86–2.03)	0.203	0.88 (0.43–1.78)	0.722
3–4 (2 score)	1.66 (1.06–2.6)	0.027^a^	1.65 (0.83–3.3)	0.150
5–6 (3 score)	2.09 (1.07–3.96)	0.026^a^	3.45 (1.42–8.1)	0.005^a^
Yi				
Not at all (0 score)	1.0 (Ref)		1.0 (Ref)	
1–2 (1 score)	2.45 (1.4–4.31)	0.002^a^	1.43 (0.7–2.91)	0.319
3–4 (2 score)	3.04 (1.64–5.67)	<0.001^a^	2.70 (1.27–5.73)	0.001^a^
5–6 (3 score)	3.28 (1.17–8.75)	0.020^a^	4.04 (1.46–10.97)	0.006^a^
Qiang				
Not at all (0 score)	1.0 (Ref)		1.0 (Ref)	
1–2 (1 score)	0.76 (0.48–1.18)	0.223	1.24 (0.51–3.07)	0.635
3–4 (2 score)	1.35 (0.86–2.12)	0.192	2.34 (0.97–5.78)	0.059
5–6 (3 score)	0.98 (0.49–1.88)	0.956	2.28 (0.71–6.77)	0.147
Uyghur				
Not at all (0 score)	1.0 (Ref)		1.0 (Ref)	
1–2 (1 score)	1.73 (0.85–3.62)	0.138	3.00 (0.38–62.45)	0.352
3–4 (2 score)	1.46 (0.69–3.18)	0.327	3.98 (0.56–80.58)	0.230
5–6 (3 score)	1.39 (0.53–3.52)	0.489	3.19 (0.34–70.65)	0.348
Others				
Not at all (0 score)	1.0 (Ref)		1.0 (Ref)	
1–2 (1 score)	2.08 (1.05–4.24)	0.039^a^	1.91 (0.74–5.13)	0.188
3–4 (2 score)	1.63 (0.72–3.65)	0.235	2.26 (0.81–6.44)	0.119
5–6 (3 score)	1.92 (0.6–5.6)	0.246	3.56 (0.94–13.43)	0.059

Model was adjusted for age, gender, educational level, marital status and chronic disease. Others = other ethnics including Zhuang, Manchu, Hui, Mongolia, Tujia ethnics.

CI, cognitive impairment; Ref, reference.

^aPearsons chi-squared test for categorical variables. Significance at p < 0.05.^

**Figure 2:**
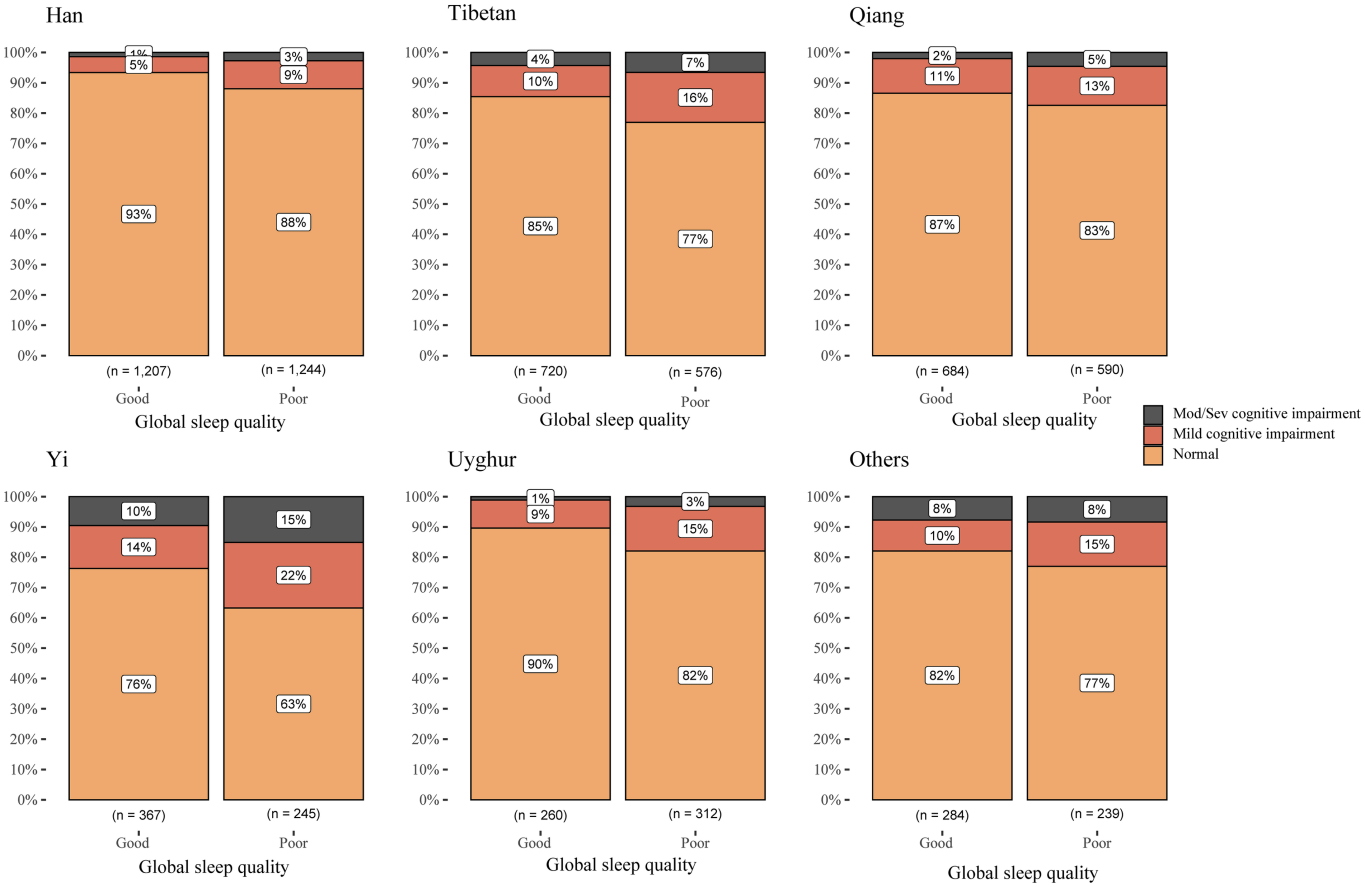
The association between sleep quality and cognition.

The highest prevalence of cognitive impairment in both the good and poor sleep quality groups is observed in the Yi ethnic group, while the lowest is in the Han ethnic group. Our study revealed that individuals with poor sleep quality exhibit a higher prevalence of cognitive impairment across all ethnic groups. Compared to individuals with good sleep quality, the proportion of cognitive impairment among Han individuals with poor sleep quality increased by 100%, representing the highest increase. This was followed by the Uyghur individuals, with an increase of 80%, the Tibetan individuals, with an increase of 64.5%, and finally, other minority ethnic groups, which had the smallest increase of only 27.8% (Figure 2).

## 4 Discussion

To the best of our knowledge, this is the first study to examine the association between sleep quality and cognitive impairment among a multi-ethnic population of middle-aged and older adults in Western China—an under-researched and underserved region. Our findings uniquely highlight the role of poor sleep quality, particularly daytime dysfunction, which showed strong associations with both mild and moderate-to-severe cognitive impairment. These results suggest that severe daytime dysfunction may be a critical risk factor for cognitive decline in this population. Notably, individuals from ethnic minority groups exhibited a significantly higher risk of cognitive impairment compared to the Han population. These insights contribute to a deeper understanding of cognitive health disparities and may inform targeted interventions in rural, multi-ethnic areas with limited healthcare resources.

Our findings, consistent with previous studies, indicate that poor sleep quality is associated with cognitive impairment, daytime dysfunction demonstrating a particularly strong correlation (29). Previous research has shown that participants with severe daytime dysfunction due to sleep problems are twice as likely to experience subjective cognitive decline compared to those without sleep disorders and daytime dysfunction (19). A systematic review and meta-analysis have also highlighted that moderate-to-high levels of evidence indicate a significant association between daytime dysfunction and an increased risk of all-cause cognitive disorders (30). In our study, we evaluated daytime dysfunction using two questions: ‘During the past month, how often have you had trouble staying awake while driving, eating meals, or engaging in social activity?’ and ‘During the past month, how much of a problem has it been for you to keep up enough enthusiasm to get things done?’ The first question addresses excessive daytime sleepiness, specifically the difficulty of staying awake during daily activities. Numerous studies have demonstrated a close association between excessive daytime sleepiness and cognitive impairment (31), suggesting that being sleepy during the day in older adults may be an early indicator of decline in cognitive functioning (32). Another cohort study demonstrated that baseline daytime sleepiness is associated with an increased risk of cognitive impairment over a 10-year period (33). The second question assesses deficits in daytime energy and motivation resulting from sleep problems. Poor nighttime sleep quality reduces vitality and motivation, adversely affecting mood and task performance (34). This decline also worsens work performance and further deteriorates sleep quality, leading to a decline in cognitive function. While prolonged sleep latency and decreased sleep efficiency are common symptoms in middle-aged and older patients with sleep disorders, these issues are not always considered serious, because they occur independently, are mild or transient, and remain within an acceptable range, thus not causing significant daytime dysfunction (35). Older adults may still perceive their overall sleep quality as satisfactory. However, when poor sleep symptoms become troublesome and lead to daytime impairments, such complaints are typically classified as sleep disturbances, indicating a severe decline in overall sleep quality (36). In line with findings from previous researches, our study also indicates that a sleep duration of approximately 7 h is optimal for maintaining cognitive function, whereas both insufficient and excessive sleep durations are associated with an increased risk of cognitive impairment (20, 37, 38).

Our study reveals significant disparities in cognitive function among various ethnic groups. After adjusting for confounding factors, the risk of mild to moderate-to-severe cognitive impairment is higher in the Tibetan, Yi, Uyghur, and other ethnic minority groups compared to the Han ethnic group. There are several reasons that may account for these differences. First, altitude differences may play a significant role. Hypoxia, or low oxygen levels, can lead to cognitive impairment and promote the progression of Alzheimer’s disease (AD) (39). Thus, the prevalence of cognitive impairment in older adults living in high altitude area is significantly higher compared to those living in low altitude area (40). In our study Tibetan, YI, Qiang, Uyghur and other ethnic minority groups reside at higher altitudes than the Han ethnic group (41). Furthermore, it has been demonstrated that dietary habits might exert a substantial impact on cognitive performance. A meta-analysis revealed a significant correlation between the consumption of total meat, fish, and poultry and the susceptibility to neurodegenerative cognitive impairment (42, 43) Tibetans follow traditional dietary practices characterized by the consumption of substantial amounts of high intake of carbohydrate (44) rice wine, buttered tea, salt, and yak meat, with limited intake of vegetables and fruit. Compared to the Han, individuals of the Yi ethnicity exhibit a higher consume of salt and have a stronger preference for sugary foods, and maintain irregular meal patterns (45). Uyghurs take great pleasure in consuming beef and mutton, often indulging in generous amounts of salt and fat (46). In our study, minority groups mainly settled in relatively remote areas or semi-pastoral regions, where their diet consisted mainly of beef and mutton. Due to relatively poor transportation conditions, they preferred pickled food, which together led to their high-fat, high-sugar, and high-salt eating habits, which are associated with increased risks of cardiovascular and cognitive disorders. Therefore, we suggest that middle-aged and older adults should avoid excessive intake of salt, fat, and sugar and increase their intake of healthy foods such as fish, shrimp, fruits, and vegetables to protect their cognitive function, especially those living in pastoral areas. Additionally, when evaluating the cognition of subjects, assessors often employ Mandarin and Chinese characters. However, ethnic minorities typically use their own languages and scripts in their daily life, which can result in underestimated cognitive assessment outcomes. In regions where Tibetan and Yi ethnic groups reside, the educational model often commonly known as the ‘first model’ is employed (47). In Tibetan-inhabited areas, this model primarily uses Tibetan as the teaching language while also incorporating Mandarin. As a result, their cognitive function assessments in Mandarin may appear lower than their actual abilities (48). Future research may employ tools like MoCA to ensure more accurate and equitable cognitive assessments in diverse populations.

Among middle age and older adults from multi-ethnic populations in western China, we found that underweight status was significantly associated with an increased risk of mild cognitive impairment, consistent with findings from other studies (49) and overweight and obesity were identified as protective factors against moderate-to-severe cognitive impairment. Although numerous studies have indicated that under the combined influence of genetic inflammatory (50) and metabolic (51) factors, lower BMI is associated with less cognitive impairment, whereas higher BMI is linked to greater cognitive decline. This relationship does not consistently apply to older adults (52). Overweight and obesity in older adults are positively associated with enhanced cognitive performance, particularly in reasoning and visuospatial abilities (53). The protective effects of higher BMI may be attributed to increased nutritional reserves, neuroprotective hormonal mechanisms, and reduced frailty (53). Another study also pointed out that underweight status (BMI < 18.5) was significantly associated with an increased risk of cognitive impairment in middle-aged and older adults, with an adjusted hazard ratio (HR) of 1.63 (95% CI: 1.12–2.37), possibly due to malnutrition or other underlying health conditions (49). Higher fat mass and lean body mass have been shown to reduce the risk of cognitive impairment, particularly in women, possibly through neuroprotective mechanisms such as greater energy reserves and hormonal regulation (54). Therefore, we suggest older people, especially the females to prevent underweight by ensuring adequate nutrition, particularly protein and essential vitamins, while avoiding excessive weight loss that could compromise muscle mass.

The correlation between daytime dysfunction and cognitive impairment is statistically significant in the overall population. However, stratified analysis by ethnicity reveals that this association is primarily observed among the Han, Tibetan, and Yi ethnic groups, with the Yi exhibiting the most pronounced effects, followed by the Tibetan. Compared to Yi individuals without daytime dysfunction, those with severe daytime dysfunction exhibit a 3.28-fold increased risk of mild cognitive impairment and a 4.04-fold increased risk of moderate to severe cognitive impairment. However, this association is not significant among the Qiang and Uyghur groups, potentially due to cultural and religious practices. The Qiang people’s traditional beliefs emphasize fearlessness and reverence for nature, promoting a submissive attitude toward fate (55). These beliefs play a crucial role in psychological regulation and maintaining social order (55–57). The majority of Uyghurs practice Islam, which offers strong spiritual support and comfort, helping them maintain a positive outlook in the face of life’s pressures and challenges. Religious practices such as prayer, fasting, and participating in religious ceremonies can alleviate anxiety and depression (58). Besides, Islam places great emphasis on community, and through collective prayers and other religious activities, Uyghur individuals strengthen social ties and cultivate a sense of belonging, ultimately enhancing their psychological well-being and overall happiness (59, 60). These factors may potentially attenuate the impact of poor sleep quality on cognitive function. Additionally, the absence of an observed correlation between daytime dysfunction and cognitive impairment among the Qiang and Uyghur groups might also be due to insufficient sample sizes for these individual ethnic groups.

Our findings indicate that 47.7% of middle-aged and older adults surveyed in western China reported poor sleep quality, suggesting that nearly half of this population experiences suboptimal sleep. Furthermore, cognitive impairment was observed in 15.3% of the participants. Based our results, we recommend that individuals aged 50 years and older to maintain an optimal sleep duration of 6–7 h (38) and prioritize addressing daytime dysfunction. Short daytime naps, ideally restricted to approximately 20 min, may serve as an effective strategy to mitigate daytime dysfunction and enhance overall cognitive health (20).

This study, characterized by a large sample size, utilized baseline data from the West-China Health and Aging Trend (WCHAT) to investigate the association between sleep and cognition. The sample encompasses multiple locations and includes participants from 10 different ethnic groups, with an in-depth analysis of the five most populous ethnic groups. The ethnic representativeness of the sample lends considerable external validity to the conclusions drawn from this study. Furthermore, we accounted for confounding factors that impact sleep and cognition, such as age, gender, educational level, marital status, and chronic diseases, thereby bolstering the reliability of our findings. Furthermore, all survey personnel received systematic and professional training, which improved the consistency and quality of data collection. Certainly, this study also has certain limitations: Firstly, the data is cross-sectional, which precludes the establishment of causal relationships between the variables. Second, the self-reported nature of sleep quality may introduce recall bias, affecting the observed association between sleep quality and cognitive impairment. Finally, although we adjusted for several relevant confounders, there may still be some potential confounders that were not measured. In the future, the WCHAT will continue to follow up with subjects annually to further explore the longitudinal relationship between changes in sleep quality and cognition impairment. We also intend to enhance objective sleep assessments, such as actigraphy or polysomnography (PSG), along with comprehensive cognitive evaluations in small community and hospital cohorts, to further investigate and validate our current research findings.

## Data Availability

The raw data supporting the conclusions of this article will be made available by the authors, without undue reservation.

## References

[1] United Nations. The 2015 revision of the UN’s world population projections. Popul Dev Rev. (2015) 41:557–61. doi: 10.1111/j.1728-4457.2015.00082.x

[2] Prince M Comas-Herrera A Knapp M Guerchet M Karagiannidou M. World Alzheimer report 2016. Improving healthcare for people living with dementia: coverage, quality and costs now and in the future. [PhD thesis] Alzheimer’s Disease International (2016).

[3] An R Liu G Khan N Yan H Wang Y. Dietary habits and cognitive impairment risk among oldest-old Chinese. J. Gerontol. Series B. (2019) 74:474–83. doi: 10.1093/geronb/gbw170, PMID: 28184889

[4] Lezak MD. Neuropsychological assessment. USA: Oxford University Press (2004).

[5] Jessen F Amariglio RE Van Boxtel M Breteler M Ceccaldi M Chételat G. A conceptual framework for research on subjective cognitive decline in preclinical Alzheimer’s disease. Alzheimers Dement. (2014) 10:844–52. doi: 10.1016/j.jalz.2014.01.001, PMID: 24798886 PMC4317324

[6] Chan KY Wang W Wu JJ Liu L Theodoratou E Car J. Epidemiology of Alzheimer’s disease and other forms of dementia in China, 1990–2010: a systematic review and analysis. Lancet. (2013) 381:2016–23. doi: 10.1016/S0140-6736(13)60221-4, PMID: 23746902

[7] Alzheimer’s Disease International. World Alzheimer Report 2019. (2020). Available at: https://www.alz.co.uk/research/world-report-2019 (Accessed September 20, 2019).

[8] Association A. 2019 Alzheimer’s disease facts and figures. Alzheimers Dement. (2019) 15:321–87. doi: 10.1016/j.jalz.2019.01.010

[9] Jia J Wei C Chen S Li F Tang Y Qin W. The cost of Alzheimer’s disease in China and re-estimation of costs worldwide. Alzheimers Dement. (2018) 14:483–91. doi: 10.1016/j.jalz.2017.12.006, PMID: 29433981

[10] Ngandu T Lehtisalo J Solomon A Levälahti E Ahtiluoto S Antikainen R. A 2 year multidomain intervention of diet, exercise, cognitive training, and vascular risk monitoring versus control to prevent cognitive decline in at-risk elderly people (FINGER): a randomised controlled trial. Lancet. (2015) 385:2255–63. doi: 10.1016/S0140-6736(15)60461-5, PMID: 25771249

[11] Livingston G Huntley J Sommerlad A Ames D Ballard C Banerjee S. Dementia prevention, intervention, and care: 2020 report of the lancet commission. Lancet. (2020) 396:413–46. doi: 10.1016/S0140-6736(20)30367-6, PMID: 32738937 PMC7392084

[12] Kim JH Duffy JF. Circadian rhythm sleep-wake disorders in older adults. Sleep Med Clin. (2018) 13:39–50. doi: 10.1016/j.jsmc.2017.09.004, PMID: 29412982

[13] Halson SL Juliff LE. Sleep, sport, and the brain. Prog Brain Res. (2017) 234:13–31. doi: 10.1016/bs.pbr.2017.06.006, PMID: 29031461

[14] Spira AP Gamaldo AA An Y Wu MN Simonsick EM Bilgel M. Self-reported sleep and β-amyloid deposition in community-dwelling older adults. JAMA Neurol. (2013) 70:1537–43. doi: 10.1001/jamaneurol.2013.4258, PMID: 24145859 PMC3918480

[15] Kang JE Lim MM Bateman RJ Lee JJ Smyth LP Cirrito JR. Amyloid-β dynamics are regulated by orexin and the sleep-wake cycle. Science. (2009) 326:1005–7. doi: 10.1126/science.1180962, PMID: 19779148 PMC2789838

[16] Fortier-Brochu E Morin CM. Cognitive impairment in individuals with insomnia: clinical significance and correlates. Sleep. (2014) 37:1787–98. doi: 10.5665/sleep.4172, PMID: 25364074 PMC4196062

[17] Zhang P Tan CW Chen GH Ge YJ Xu J Xia L. Patients with chronic insomnia disorder have increased serum levels of neurofilaments, neuron-specific enolase and S100B: does organic brain damage exist? Sleep Med. (2018) 48:163–71. doi: 10.1016/j.sleep.2017.12.012, PMID: 29957487

[18] Joo EY Noh HJ Kim JS Koo DL Kim D Hwang KJ. Brain gray matter deficits in patients with chronic primary insomnia. Sleep. (2013) 36:999–1007. doi: 10.5665/sleep.2796, PMID: 23814336 PMC3669067

[19] Kim JH Ahn JH Min CY Yoo DM Choi HG. Association between sleep quality and subjective cognitive decline: evidence from a community health survey. Sleep Med. (2021) 83:123–31. doi: 10.1016/j.sleep.2021.04.031, PMID: 33993029

[20] Ma Y Liang L Zheng F Shi L Zhong B Xie W. Association between sleep duration and cognitive decline. JAMA Netw Open. (2020) 3:e2013573–3. doi: 10.1001/jamanetworkopen.2020.13573, PMID: 32955572 PMC7506513

[21] Ma XQ Jiang CQ Xu L Zhang WS Zhu F Jin YL. Sleep quality and cognitive impairment in older Chinese: Guangzhou biobank cohort study. Age Ageing. (2020) 49:119–24. doi: 10.1093/ageing/afz120, PMID: 31665199

[22] Chen R Wang J Pederson AM Prather AA Hirst AK Ackley S. Evaluation of racial and ethnic heterogeneity in the associations of sleep quality and sleep apnea risk with cognitive function and cognitive decline. Alzheimers Dementia. (2024) 10:e12441. doi: 10.1002/trc2.12441, PMID: 38356481 PMC10865460

[23] National Bureau of Statistics of China. Bulletin of the seventh national population census. (2021). Available at: http://www.stats.gov.cn/english/PressRelease/202105/t20210510_1817185.html (Accessed May 11, 2021).

[24] Zhou Z Fang Y Zhou Z Li D Wang D Li Y. Assessing income-related health inequality and horizontal inequity in China. Soc Indic Res. (2017) 132:241–56. doi: 10.1007/s11205-015-1221-1

[25] National Bureau of Statistics of China. China population census yearbook 2020. Beijing: China Statistics Press (2021).

[26] Buysse DJ Reynolds CF III Monk TH Berman SR Kupfer DJ. The Pittsburgh sleep quality index: a new instrument for psychiatric practice and research. Psychiatry Res. (1989) 28:193–213. doi: 10.1016/0165-1781(89)90047-4, PMID: 2748771

[27] Pfeiffer E. A short portable mental status questionnaire for the assessment of organic brain deficit in elderly patients. J Am Geriatr Soc. (1975) 23:433–41. doi: 10.1111/j.1532-5415.1975.tb00927.x, PMID: 1159263

[28] China NHC. Guidelines for the diagnosis and treatment of obesity (2024 edition). Beijing: National Health Commission of China (2024).

[29] Henneghan AM Carter P Stuifbergan A Parmelee B Kesler S. Relationships between self-reported sleep quality components and cognitive functioning in breast cancer survivors up to 10 years following chemotherapy. Psycho Oncol. (2018) 27:1937–43. doi: 10.1002/pon.4745, PMID: 29683228

[30] Xu W Tan CC Zou JJ Cao XP Tan L. Sleep problems and risk of all-cause cognitive decline or dementia: an updated systematic review and meta-analysis. J Neurol Neurosurg Psychiatry. (2020) 91:236–44. doi: 10.1136/jnnp-2019-321896, PMID: 31879285 PMC7035682

[31] Merlino G Piani A Gigli GL Cancelli I Rinaldi A Baroselli A. Daytime sleepiness is associated with dementia and cognitive decline in older Italian adults: a population-based study. Sleep Med. (2010) 11:372–7. doi: 10.1016/j.sleep.2009.07.018, PMID: 20219426

[32] Foley D Monjan A Masaki K Ross W Havlik R White L. Daytime sleepiness is associated with 3-year incident dementia and cognitive decline in older Japanese-American men. J Am Geriatr Soc. (2001) 49:1628–32. doi: 10.1046/j.1532-5415.2001.t01-1-49271.x PMID: 11843995

[33] Keage HAD Banks S Yang KL Morgan K Brayne C Matthews FE. What sleep characteristics predict cognitive decline in the elderly? Sleep Med. (2012) 13:886–92. doi: 10.1016/j.sleep.2012.02.003, PMID: 22560827

[34] Dinges DF Pack F Williams K Gillen KA Powell JW Ott GE. Cumulative sleepiness, mood disturbance, and psychomotor vigilance performance decrements during a week of sleep restricted to 4–5 hours per night. Sleep. (1997) 20:267–77. PMID: 9231952

[35] Li J Vitiello MV Gooneratne NS. Sleep in normal aging. Sleep Med Clin. (2022) 17:161–71. doi: 10.1016/j.jsmc.2022.02.007, PMID: 35659071

[36] Ohayon MM. Epidemiology of insomnia: what we know and what we still need to learn. Sleep Med Rev. (2002) 6:97–111. doi: 10.1053/smrv.2002.0186 PMID: 12531146

[37] Li Y Sahakian BJ Kang J Langley C Zhang W Xie C. The brain structure and genetic mechanisms underlying the non-linear association between sleep duration, cognition and mental health. Nature Aging. (2022) 2:425–37. doi: 10.1038/s43587-022-00210-2, PMID: 37118065

[38] Bloomberg M Brocklebank L Hamer M Steptoe A. Joint associations of physical activity and sleep duration with cognitive ageing: longitudinal analysis of an English cohort study. Lancet Healthy Longevity. (2023) 4:e345–53. doi: 10.1016/S2666-7568(23)00083-1, PMID: 37421962 PMC11883718

[39] Zhang X Le W. Pathological role of hypoxia in Alzheimer’s disease. Exp Neurol. (2010) 223:299–303. doi: 10.1016/j.expneurol.2009.07.033 19679125

[40] Liu S Wang F Zhang C Zhang Q Dang ZC Ng CH. Cognitive impairment and its associated factors in older adults living in high and low altitude areas: a comparative study. Front Psychiatry. (2022) 13:1414. doi: 10.3389/fpsyt.2022.871414, PMID: 35815014 PMC9259941

[41] Zhao Y Lu Y Zhao W Wang Y Ge M Zhou L. Long sleep duration is associated with cognitive frailty among older community-dwelling adults: results from West China health and aging trend study. BMC Geriatr. (2021) 21:608. doi: 10.1186/s12877-021-02455-9, PMID: 34706663 PMC8555015

[42] Granic A Davies K Adamson A Kirkwood T Hill TR Siervo M. Dietary patterns high in red meat, potato, gravy, and butter are associated with poor cognitive functioning but not with rate of cognitive decline in very old adults. J Nutr. (2016) 146:265–74. doi: 10.3945/jn.115.216952, PMID: 26740685 PMC4725429

[43] Quan W Xu Y Luo J Zeng M He Z Shen Q. Association of dietary meat consumption habits with neurodegenerative cognitive impairment: an updated systematic review and dose–response meta-analysis of 24 prospective cohort studies. Food Funct. (2022) 13:12590–601. doi: 10.1039/D2FO03168J, PMID: 36385382

[44] Liu T. A preliminary study on diet adaptation mechanism of Tibetan population [Master’s thesis]. Dali, China: Yunnan University (2023).

[45] Xu H Ling L Xia P Ruiyu Q. A survey and analysis of the lifestyle habits of healthy adults of the Yi ethnic group in Yunnan. Chin J Health Manag. (2013) 7:333–4.

[46] Li T Shu X Qiangqiang Y Caihong L Guilt R Xiong L. Investigation on diet and nutritional status of 515 Uyghur residents. J Xinjiang Med Univ. (2017) 40:523–5.

[47] Qupi L. Study on difficulties and solutions for type one teaching mode in Tibetan region of Sichuan [Master’s Thesis]. Sichuan Normal University (2014).

[48] Renqing T Dawa SM. A survey on the standard spoken and written Chinese language ability of Tibetan primary school teachers in Tibet and its influencing factors. J Xizhang Univ. (2023) 38:241–8.

[49] Xiang X An R. Body weight status and onset of cognitive impairment among US middle-aged and older adults. Arch Gerontol Geriatr. (2015) 60:394–400. doi: 10.1016/j.archger.2015.02.008, PMID: 25747849

[50] Le Thuc O García-Cáceres C. Obesity-induced inflammation: connecting the periphery to the brain. Nat Metab. (2024) 6:1237–52. doi: 10.1038/s42255-024-01079-8, PMID: 38997442

[51] Kouvari M D’cunha NM Travica N Sergi D Zec M Marx W. Metabolic syndrome, cognitive impairment and the role of diet: a narrative review. Nutrients. (2022) 14:333. doi: 10.3390/nu14020333, PMID: 35057514 PMC8780484

[52] Dye L Boyle NB Champ C Lawton C. The relationship between obesity and cognitive health and decline. Proc Nutr Soc. (2017) 76:443–54. doi: 10.1017/S0029665117002014, PMID: 28889822

[53] Kuo HK Jones RN Milberg WP Tennstedt S Talbot L Morris JN. Cognitive function in normal-weight, overweight, and obese older adults: an analysis of the advanced cognitive training for independent and vital elderly cohort. J Am Geriatr Soc. (2006) 54:97–103. doi: 10.1111/j.1532-5415.2005.00522.x, PMID: 16420204 PMC2834231

[54] Noh HM Oh S Song HJ Lee EY Jeong JY Ryu OH. Relationships between cognitive function and body composition among community-dwelling older adults: a cross-sectional study. BMC Geriatr. (2017) 17:259. doi: 10.1186/s12877-017-0651-9, PMID: 29096612 PMC5667483

[55] Xiao Y. Qiang folk beliefs and their social value functions. J Southwest Univ Nation. (2015) 36:41–6.

[56] Han L Berry JW Zheng Y. The relationship of acculturation strategies to resilience: the moderating impact of social support among Qiang ethnicity following the 2008 Chinese earthquake. PLoS One. (2016) 11:e0164484. doi: 10.1371/journal.pone.0164484, PMID: 27741274 PMC5065190

[57] Fan ZF Yu GL Liu CH. A literature review of psychological suffering: focusing on the psychological suffering of the people affected by the may 12 Wenchuan earthquake. Adv Psychol Sci. (2009) 17:631–8.

[58] Saged AAG Sa’ari CZ Bin AM Al-Rahmi WM Ismail WM MIA Z. The effect of an Islamic-based intervention on depression and anxiety in Malaysia. J Relig Health. (2022) 61:79–92. doi: 10.1007/s10943-021-01484-3, PMID: 34981449 PMC8722650

[59] Seixas AA Briggs AQ Blanc J Moore J Chung A Williams E. Sleep health among racial/ethnic groups and strategies to achieve sleep health equity In: Essentials of sleep medicine: a practical approach to patients with sleep complaints. Cham, Switzerland: Springer Nature (2022). 47–68.

[60] Significance of Hajj for Individuals and the Community [Internet]. (2024). Available online at: https://fiqh.islamonline.net/en/significance-of-hajj-for-individuals-and-the-community/ (Accessed June 20, 2024).

