# Supplementary material for: The association between sleep quality and cognitive impairment among a multi-ethnic population of middle-aged and older adults in Western China: a multi-center cross-sectional study
Source: Front Public Health. 2025 Apr 17;13:1500027. doi: 10.3389/fpubh.2025.1500027 (PMC12043481; doi:10.3389/fpubh.2025.1500027)
Supplement: Supplementary file 1 [file Data_Sheet_1.docx]

**SUPPLEMENTARY**

**TABLE S1** Associations between subjective sleep quality and cognitive impairment across ethnic groups.

**TABLE S2** Associations between sleep latency and cognitive impairment across ethnic groups.

**TABLE S3** Associations between sleep duration and cognitive impairment across ethnic groups.

**TABLE S4** Associations between sleep efficiency and cognitive impairment across ethnic groups.

**TABLE S5** Associations between sleep disturbances and cognitive impairment across ethnic groups.

**TABLE S6** Associations between hypnotic drugs and cognitive impairment across ethnic groups.

**TABLE S7** Characteristics of the participants according to sleep quality.

**TABLE S1** Associations between subjective sleep quality and cognitive impairment across ethnic groups.

| Subjective sleep quality | Mild CI, OR [95%CI] | p value | Mod/Sev CI, OR [95%CI] | p value |
| --- | --- | --- | --- | --- |
| Han |  |  |  |  |
| Very good (0 score) | 1.0 (Ref) |  | 1.0 (Ref) |  |
| Fairly good (1 score) | 1.37 (0.82 - 2.41) | 0.249 ^a^ | 1.10 (0.43 - 3.42) | 0.850 |
| Fairly bad (2 score) | 1.90 (1.11 - 3.41) | 0.024 ^a^ | 2.65 (1.05 - 8.12) | 0.057 |
| Very bad (3 score) | 1.29 (0.58 - 2.8) | 0.520 | 1.38 (0.32 - 5.54) | 0.646 |
| Tibetan |  |  |  |  |
| Very good (0 score) | 1.0 (Ref) |  | 1.0 (Ref) |  |
| Fairly good (1 score) | 1.07 (0.63 - 1.89) | 0.811 | 0.98 (0.41 - 2.71) | 0.965 |
| Fairly bad (2 score) | 1.08 (0.6 - 2) | 0.812 | 1.30 (0.52 - 3.75) | 0.593 |
| Very bad (3 score) | 0.14 (0.01 - 0.75) | 0.063 | 0.28 (0.01 - 1.87) | 0.262 |
| Yi |  |  |  |  |
| Very good (0 score) | 1.0 (Ref) |  | 1.0 (Ref) |  |
| Fairly good (1 score) | 0.65 (0.37 - 1.13) | 0.121 | 1.89 (0.85 - 4.64) | 0.138 |
| Fairly bad (2 score) | 0.80 (0.41 - 1.56) | 0.513 | 2.46 (1 - 6.49) | 0.056 |
| Very bad (3 score) | 0.97 (0.27 - 3.11) | 0.959 | 1.85 (0.35 - 8.03) | 0.429 |
| Qiang |  |  |  |  |
| Very good (0 score) | 1.0 (Ref) |  | 1.0 (Ref) |  |
| Fairly good (1 score) | 0.94 (0.59 - 1.51) | 0.780 | 1.45 (0.56 - 4.53) | 0.479 |
| Fairly bad (2 score) | 0.83 (0.48 - 1.45) | 0.522 | 1.77 (0.61 - 5.9) | 0.314 |
| Very bad (3 score) | 1.12 (0.41 - 2.76) | 0.819 | 2.41 (0.44 - 11.24) | 0.270 |
| Uyghur |  |  |  |  |
| Very good (0 score) | 1.0 (Ref) |  | 1.0 (Ref) |  |
| Fairly good (1 score) | 2.21 (1.04 - 5.14) | 0.049 ^a^ | 1.75 (0.23 - 35.77) | 0.629 |
| Fairly bad (2 score) | 2.03 (0.89 - 4.95) | 0.102 | 2.00 (0.26 - 41.41) | 0.554 |
| Very bad (3 score) | 2.74 (0.65 - 9.97) | 0.139 | 18.53(2.04-415.41) | 0.019^a^ |
| Others |  |  |  |  |
| Very good (0 score) | 1.0 (Ref) |  | 1.0 (Ref) |  |
| Fairly good (1 score) | 2.26 (0.93 - 6.39) | 0.092 | 1.24 (0.43 - 3.97) | 0.696 |
| Fairly bad (2 score) | 2.30 (0.87 - 6.89) | 0.110 | 0.83 (0.26 - 2.88) | 0.765 |
| Very bad (3 score) | 2.15 (0.53 - 8.42) | 0.268 | 0.52 (0.06 - 2.97) | 0.488 |

Model was adjusted for age,gender,educational level,marriage status and chronic disease. Others = other ethnics including Zhuang, Manchu, Hui, Mongolia, Tujia ethnics.

CI: Cognitive impairment; Ref: Reference.

^a^ Pearsons chi-squared test for categorical variables. Significance at p < 0.05.

**TABLE S2** Associations between sleep latency and cognitive impairment across ethnic groups.

| Sleep latency | Mild CI, OR [95%CI] | p value | Mod/Sev CI, OR [95%CI] | p value |
| --- | --- | --- | --- | --- |
| Han |  |  |  |  |
| 0 h (0 score) | 1.0 (Ref) |  | 1.0 (Ref) |  |
| 1-2(1 score) | 0.89 (0.56 - 1.42) | 0.629 | 0.76 (0.32 - 1.83) | 0.541 |
| 3-4 (2 score) | 1.54 (0.99 - 2.41) | 0.059 | 1.08 (0.44 - 2.64) | 0.859 |
| 5-6 (3 score) | 1.26 (0.78 - 2.04) | 0.342 | 1.53 (0.69 - 3.55) | 0.303 |
| Tibetan |  |  |  |  |
| 0 h (0 score) | 1.0 (Ref) |  | 1.0 (Ref) |  |
| 1-2(1 score) | 1.49 (0.95 - 2.35) | 0.082 | 1.21 (0.61 - 2.45) | 0.591 |
| 3-4 (2 score) | 1.87 (1.16 - 3.03) | 0.010 ^a^ | 1.46 (0.69 - 3.08) | 0.319 |
| 5-6 (3 score) | 1.56 (0.87 - 2.75) | 0.129 | 1.02 (0.4 - 2.47) | 0.962 |
| Yi |  |  |  |  |
| 0 h (0 score) | 1.0 (Ref) |  | 1.0 (Ref) |  |
| 1-2(1 score) | 1.74 (0.98 - 3.14) | 0.061 | 0.84 (0.41 - 1.69) | 0.619 |
| 3-4 (2 score) | 1.79 (0.9 - 3.53) | 0.092 | 1.51 (0.73 - 3.14) | 0.265 |
| 5-6 (3 score) | 2.07 (1.02 - 4.21) | 0.044 ^a^ | 0.82 (0.32 - 1.98) | 0.673 |
| Qiang |  |  |  |  |
| 0 h (0 score) | 1.0 (Ref) |  | 1.0 (Ref) |  |
| 1-2(1 score) | 0.84 (0.52 - 1.36) | 0.469 | 0.58 (0.19 - 1.71) | 0.323 |
| 3-4 (2 score) | 0.76 (0.45 - 1.28) | 0.304 | 2.44 (1.03 - 6.27) | 0.050 |
| 5-6 (3 score) | 1.27 (0.76 - 2.11) | 0.360 | 1.07 (0.35 - 3.18) | 0.901 |
| Uyghur |  |  |  |  |
| 0 h (0 score) | 1.0 (Ref) |  | 1.0 (Ref) |  |
| 1-2(1 score) | 0.98 (0.48 - 1.97) | 0.950 | 1.29 (0.15 - 11.28) | 0.808 |
| 3-4 (2 score) | 1.16 (0.52 - 2.51) | 0.706 | 1.37 (0.15 - 12.25) | 0.766 |
| 5-6 (3 score) | 1.05 (0.51 - 2.15) | 0.896 | 2.71 (0.57 - 19.7) | 0.247 |
| Others |  |  |  |  |
| 0 h (0 score) | 1.0 (Ref) |  | 1.0 (Ref) |  |
| 1-2(1 score) | 0.99 (0.46 - 2.16) | 0.987 | 2.16 (0.82 - 6.09) | 0.127 |
| 3-4 (2 score) | 2.09 (0.99 - 4.48) | 0.055 | 1.39 (0.43 - 4.47) | 0.575 |
| 5-6 (3 score) | 1.05 (0.41 - 2.59) | 0.911 | 1.53 (0.47 - 4.92) | 0.474 |

Model was adjusted for age,gender,educational level,marriage status and chronic disease. Others = other ethnics including Zhuang, Manchu, Hui, Mongolia, Tujia ethnics.

CI: Cognitive impairment; Ref: Reference.

^a^ Pearsons chi-squared test for categorical variables. Significance at p < 0.05.

**TABLE S3** Associations between sleep duration and cognitive impairment across ethnic groups.

| Sleep duration | Mild CI, OR [95%CI] | p value | Mod/Sev CI, OR [95%CI] | p value |
| --- | --- | --- | --- | --- |
| Han |  |  |  |  |
| ≥8h (0 score) | 1.0 (Ref) |  | 1.0 (Ref) |  |
| 6-7h(1 score) | 0.71 (0.46 - 1.08) | 0.117 | 0.68 (0.3 - 1.41) | 0.323 |
| 5-6h (2 score) | 0.91 (0.6 - 1.35) | 0.646 | 0.85 (0.4 - 1.72) | 0.664 |
| <5h (3 score) | 0.82 (0.41 - 1.51) | 0.544 | 0.36 (0.06 - 1.3) | 0.183 |
| Tibetan |  |  |  |  |
| ≥8h (0 score) | 1.0 (Ref) |  | 1.0 (Ref) |  |
| 6-7h(1 score) | 0.85 (0.53 - 1.35) | 0.509 | 0.64 (0.27 - 1.36) | 0.278 |
| 5-6h (2 score) | 0.93 (0.56 - 1.5) | 0.777 | 1.08 (0.52 - 2.1) | 0.837 |
| <5h (3 score) | 0.74 (0.17 - 2.28) | 0.640 | 0.75 (0.04 - 4.05) | 0.790 |
| Yi | 0.85 (0.53 - 1.35) | 0.509 | 0.64 (0.27 - 1.36) | 0.278 |
| ≥8h (0 score) | 1.0 (Ref) |  | 1.0 (Ref) |  |
| 6-7h(1 score) | 0.83 (0.46 - 1.46) | 0.527 | 0.73 (0.34 - 1.48) | 0.399 |
| 5-6h (2 score) | 0.78 (0.38 - 1.5) | 0.469 | 1.53 (0.75 - 3.06) | 0.236 |
| <5h (3 score) | 1.59 (0.55 - 4.35) | 0.372 | 0.34 (0.02 - 2.1) | 0.334 |
| Qiang |  |  |  |  |
| ≥8h (0 score) | 1.0 (Ref) |  | 1.0 (Ref) |  |
| 6-7h(1 score) | 0.65 (0.41 - 1.02) | 0.071 | 1.28 (0.59 - 2.64) | 0.520 |
| 5-6h (2 score) | 0.59 (0.33 - 1.01) | 0.065 | 0.86 (0.28 - 2.21) | 0.765 |
| <5h (3 score) | 0.41 (0.10 - 1.20) | 0.153 | N/A | 0.992 |
| Uyghur |  |  |  |  |
| ≥8h (0 score) | 1.0 (Ref) |  | 1.0 (Ref) |  |
| 6-7h(1 score) | 1.59 (0.82 - 3.1) | 0.168 | 0.56 (0.07 - 2.98) | 0.524 |
| 5-6h (2 score) | 1.71 (0.86 - 3.37) | 0.123 | 0.33 (0.04 - 1.92) | 0.250 |
| <5h (3 score) | 2.43 (0.63 - 7.8) | 0.157 | 6.82 (1.25 - 37.9) | 0.025^a^ |
| Others |  |  |  |  |
| ≥8h (0 score) | 1.0 (Ref) |  | 1.0 (Ref) |  |
| 6-7h(1 score) | 0.78 (0.37 - 1.59) | 0.510 | 0.35 (0.11 - 0.98) | 0.060 |
| 5-6h (2 score) | 1.45 (0.7 - 2.94) | 0.305 | 0.81 (0.29 - 2.12) | 0.677 |
| <5h (3 score) | 0.17 (0.01 - 0.9) | 0.093 | 0.14 (0.01 - 0.87) | 0.080 |

Model was adjusted for age,gender,educational level,marriage status and chronic disease. Others = other ethnics including Zhuang, Manchu, Hui, Mongolia, Tujia ethnics.

CI: Cognitive impairment; Ref: Reference.

^a^ Pearsons chi-squared test for categorical variables. Significance at p < 0.05.

**TABLE S4** Associations between sleep efficiency and cognitive impairment across ethnic groups.

| Sleep efficiency | Mild CI, OR [95%CI] | p value | Mod/Sev CI, OR [95%CI] | p value |
| --- | --- | --- | --- | --- |
| Han |  |  |  |  |
| ≥85% (0 score) | 1.0 (Ref) |  | 1.0 (Ref) |  |
| 75-84% (1 score) | 0.82 (0.52 - 1.27) | 0.392 | 1.29 (0.6 - 2.6) | 0.498 |
| 65-74% (2 score) | 0.68 (0.33 - 1.26) | 0.251 | 1.03 (0.33 - 2.58) | 0.959 |
| <65% (3 score) | 0.96 (0.59 - 1.53) | 0.881 | 0.62 (0.2 - 1.53) | 0.339 |
| Tibetan |  |  |  |  |
| ≥85% (0 score) | 1.0 (Ref) |  | 1.0 (Ref) |  |
| 75-84% (1 score) | 1.08 (0.67 - 1.71) | 0.741 | 0.95 (0.43 - 1.92) | 0.897 |
| 65-74% (2 score) | 1.47 (0.73 - 2.81) | 0.260 | 1.32 (0.42 - 3.37) | 0.597 |
| <65% (3 score) | 1.21 (0.63 - 2.19) | 0.554 | 0.92 (0.3 - 2.32) | 0.870 |
| Yi |  |  |  |  |
| ≥85% (0 score) | 1.0 (Ref) |  | 1.0 (Ref) |  |
| 75-84% (1 score) | 1.42 (0.71 - 2.75) | 0.309 | 1.4 (0.6 - 3.06) | 0.420 |
| 65-74% (2 score) | 1.45 (0.48 - 3.9) | 0.477 | 0.61 (0.09 - 2.47) | 0.540 |
| <65% (3 score) | 0.8 (0.32 - 1.83) | 0.614 | 0.86 (0.31 - 2.17) | 0.761 |
| Qiang |  |  |  |  |
| ≥85% (0 score) | 1.0 (Ref) |  | 1.0 (Ref) |  |
| 75-84% (1 score) | 1.19 (0.75 - 1.86) | 0.451 | 1.32 (0.58 - 2.78) | 0.485 |
| 65-74% (2 score) | 1.01 (0.49 - 1.92) | 0.976 | 0.3 (0.02 - 1.51) | 0.246 |
| <65% (3 score) | 0.72 (0.32 - 1.45) | 0.395 | 0.28 (0.02 - 1.41) | 0.220 |
| Uyghur |  |  |  |  |
| ≥85% (0 score) | 1.0 (Ref) |  | 1.0 (Ref) |  |
| 75-84% (1 score) | 0.78 (0.22 - 2.13) | 0.658 | 1.61 (0.21 - 7.91) | 0.589 |
| 65-74% (2 score) | 0.35 (0.02 - 1.88) | 0.322 | N/A | 0.993 |
| <65% (3 score) | 1.87 (0.97 - 3.52) | 0.057 | 2.81 (0.66 - 11.05) | 0.139 |
| Others |  |  |  |  |
| ≥85% (0 score) | 1.0 (Ref) |  | 1.0 (Ref) |  |
| 75-84% (1 score) | 0.66 (0.21 - 1.7) | 0.423 | 1.79 (0.55 - 5.36) | 0.311 |
| 65-74% (2 score) | 1.01 (0.37 - 2.44) | 0.980 | 0.53 (0.11 - 1.87) | 0.366 |
| <65% (3 score) | 0.47 (0.15 - 1.2) | 0.145 | 0.5 (0.13 - 1.61) | 0.280 |

Model was adjusted for age,gender,educational level,marriage status and chronic disease. Others = other ethnics including Zhuang, Manchu, Hui, Mongolia, Tujia ethnics.

CI: Cognitive impairment; Ref: Reference.

**TABLE S5** Associations between sleep disturbances and cognitive impairment across ethnic groups.

| Sleep disturbances | Mild CI, OR [95%CI] | p value | Mod/Sev CI, OR [95%CI] | p value |
| --- | --- | --- | --- | --- |
| Han |  |  |  |  |
| Not at all (0 score) | 1.0 (Ref) |  | 1.0 (Ref) |  |
| 1-9 (1 score) | 1.44 (0.57 - 4.88) | 0.494 | N/A | 0.982 |
| 10-18 (2 score) | 2.21 (0.85 - 7.56) | 0.144 | N/A | 0.982 |
| 19-28 (3 score) | 1.18 (0.15 - 6.84) | 0.860 | N/A | 0.980 |
| Tibetan |  |  |  |  |
| Not at all (0 score) | 1.0 (Ref) |  | 1.0 (Ref) |  |
| 1-9 (1 score) | 1.52 (0.58 - 5.24) | 0.447 | 1.08 (0.29 - 7.03) | 0.924 |
| 10-18 (2 score) | 2.44 (0.91 - 8.53) | 0.109 | 1.41 (0.36 - 9.4) | 0.664 |
| 19-28 (3 score) | 1.5 (0.18 - 9.28) | 0.673 | 1.74 (0.17 - 17.58) | 0.622 |
| Yi |  |  |  |  |
| Not at all (0 score) | 1.0 (Ref) |  | 1.0 (Ref) |  |
| 1-9 (1 score) | 0.95 (0.31 - 3.57) | 0.933 | 1.13 (0.29 - 6.11) | 0.876 |
| 10-18 (2 score) | 1.91 (0.6 - 7.51) | 0.305 | 3.1 (0.76 - 17.58) | 0.149 |
| 19-28 (3 score) | 2.45 (0.23 - 24.43) | 0.436 | 7.89 (0.76 - 99.46) | 0.091 |
| Qiang |  |  |  |  |
| Not at all (0 score) | 1.0 (Ref) |  | 1.0 (Ref) |  |
| 1-9 (1 score) | 0.42 (0.16 - 1.35) | 0.108 | 0.63 (0.1 - 12.3) | 0.679 |
| 10-18 (2 score) | 0.4 (0.14 - 1.29) | 0.093 | 0.61 (0.1 - 12.07) | 0.662 |
| 19-28 (3 score) | 1.47 (0.4 - 5.92) | 0.573 | 1.15 (0.08 - 29.65) | 0.919 |
| Uyghur |  |  |  |  |
| Not at all (0 score) | 1.0 (Ref) |  | 1.0 (Ref) |  |
| 1-9 (1 score) | N/A | 0.983 | N/A | 0.990 |
| 10-18 (2 score) | N/A | 0.983 | N/A | 0.990 |
| 19-28 (3 score) | N/A | 0.983 | N/A | 0.990 |
| Others |  |  |  |  |
| Not at all (0 score) | 1.0 (Ref) |  | 1.0 (Ref) |  |
| 1-9 (1 score) | 0.44 (0.15 - 1.41) | 0.147 | 0.21 (0.05 - 0.88) | 0.030 ^a^ |
| 10-18 (2 score) | 0.27 (0.08 - 0.97) | 0.039^a^ | 0.12 (0.02 - 0.55) | 0.007 ^a^ |
| 19-28 (3 score) | N/A | 0.983 | 1.39 (0.04 - 33.64) | 0.845 |

Model was adjusted for age,gender,educational level,marriage status and chronic disease. Others = other ethnics including Zhuang, Manchu, Hui, Mongolia, Tujia ethnics.

CI: Cognitive impairment; Ref: Reference.

^a^ Pearsons chi-squared test for categorical variables. Significance at p < 0.05.

**TABLE S6** Associations between hypnotic drugs and cognitive impairment across ethnic groups.

| Hypnotic drugs | Mild CI, OR [95%CI] | p value | Mod/Sev CI, OR [95%CI] | p value |
| --- | --- | --- | --- | --- |
| Han |  |  |  |  |
| Not during the last month (0 score) | 1.0 (Ref) |  | 1.0 (Ref) |  |
| < 1time per week (1 score) | 1.02 (0.16 - 3.65) | 0.974 | 2.26 (0.12 - 12.47) | 0.447 |
| 1-2 time per week (2 score) | 0.72 (0.04 - 3.73) | 0.752 | N/A | 0.988 |
| ≥ 3 per week (3 score) | 0.33 (0.02 - 1.67) | 0.287 | 2.69 (0.39 - 10.88) | 0.221 |
| Tibetan |  |  |  |  |
| Not during the last month (0 score) | 1.0 (Ref) |  | 1.0 (Ref) |  |
| < 1time per week (1 score) | 0.91 (0.14 - 3.36) | 0.902 | N/A | 0.992 |
| 1-2 time per week (2 score) | 0.66 (0.04 - 3.59) | 0.697 | N/A | 0.994 |
| ≥ 3 per week (3 score) | 1.15 (0.17 - 4.73) | 0.866 | 3.45 (0.5 - 15.15) | 0.135 |
| Yi |  |  |  |  |
| Not during the last month (0 score) | 1.0 (Ref) |  | 1.0 (Ref) |  |
| < 1time per week (1 score) | 5.81 (0.2 - 168.58) | 0.245 | N/A | 0.990 |
| 1-2 time per week (2 score) | 0 (NA - Inf) | 0.997 | N/A | 0.988 |
| ≥ 3 per week (3 score) | 3.39 (0.13 - 89.54) | 0.400 | 1.61 (0.07 - 19.38) | 0.709 |
| Qiang |  |  |  |  |
| Not during the last month (0 score) | 1.0 (Ref) |  | 1.0 (Ref) |  |
| < 1time per week (1 score) | 0.39 (0.02 - 2.06) | 0.369 | N/A | 0.995 |
| 1-2 time per week (2 score) | 0.62 (0.03 - 3.51) | 0.658 | N/A | 0.996 |
| ≥ 3 per week (3 score) | 3.05 (0.58 - 13.55) | 0.150 | N/A | 0.997 |
| Uyghur |  |  |  |  |
| Not during the last month (0 score) | 1.0 (Ref) |  | 1.0 (Ref) |  |
| < 1time per week (1 score) | 2.76 (0.55 - 11.27) | 0.172 | N/A | 0.992 |
| 1-2 time per week (2 score) | N/A | 0.989 | N/A | 0.995 |
| ≥ 3 per week (3 score) | N/A | 0.987 | N/A | 0.996 |
| Others |  |  |  |  |
| Not during the last month (0 score) | 1.0 (Ref) |  | 1.0 (Ref) |  |
| < 1time per week (1 score) | N/A | 0.987 | N/A | 0.992 |
| 1-2 time per week (2 score) | 1.19 (0.06 - 8.39) | 0.879 | N/A | 0.991 |
| ≥ 3 per week (3 score) | 2.87 (0.31 - 17.49) | 0.288 | N/A | 0.994 |

Model was adjusted for age, gender, educational level, marriage status and chronic disease. Others = other ethnics including Zhuang, Manchu, Hui, Mongolia, Tujia ethnics.

CI: Cognitive impairment; Ref: Reference.

Table S7Characteristics of the participants according to sleep quality.

| Characters | Total | good sleep quality | poor sleep quality | p value |
| --- | --- | --- | --- | --- |
| Total Number, n | 6728 | 3522 | 3206 |  |
| Age (mean (SD)) | 62.39 (8.25) | 61.87 (8.10) | 62.96 (8.38) | <0.001^a^ |
| BMI (mean (SD)) | 25.34 (4.19) | 25.30 (4.00) | 25.40 (4.30) | 0.346 |
| Cognition score (mean (SD)) | 1.13 (1.49) | 1.01 (1.41) | 1.25 (1.57) | <0.001^a^ |
| Cognition score |  |  |  |  |
| Normal | 5700 (84.70) | 3080 (87.50) | 2620 (81.70) | <0.001^b^ |
| Mild CI | 740 (11.00) | 320 (9.10) | 420 (13.10) |  |
| Mod/Sev CI | 288 (4.30) | 122 (3.50) | 166 (5.20) |  |
| Chronic Diseases (%) |  |  |  | <0.001^b^ |
| No | 3680 (54.70) | 2129 (60.40) | 1551 (48.40) |  |
| Yes | 3048 (45.30) | 1393 (39.60) | 1655 (51.60) |  |
| Ethic (%) |  |  |  | <0.001^b^ |
| Han | 2451 (36.40) | 1207 (34.30) | 1244 (38.80) |  |
| Tibetan | 1296 (19.30) | 720 (20.40) | 576 (18.00) |  |
| Qiang | 1274 (18.90) | 684 (19.40) | 590 (18.40) |  |
| Yi | 612 (9.10) | 367 (10.40) | 245 (7.60) |  |
| Uyghur | 572 (8.50) | 260 (7.40) | 312 (9.70) |  |
| Others | 523 (7.80) | 284 (8.10) | 239 (7.50) |  |
| Gender (%) |  |  |  | <0.001^b^ |
| Male | 2520 (37.50) | 1600 (45.40) | 920 (28.70) |  |
| Female | 4208 (62.50) | 1922 (54.60) | 2286 (71.30) |  |
| Educational level (%) |  |  |  | <0.001^b^ |
| No formal education | 1859 (27.60) | 858 (24.40) | 1001 (31.20) |  |
| Elementary school | 2283 (33.90) | 1159 (32.90) | 1124 (35.10) |  |
| Middle school | 1453 (21.60) | 833 (23.70) | 620 (19.30) |  |
| High school | 413 (6.10) | 240 (6.80) | 173 (5.40) |  |
| High above | 720 (10.70) | 432 (12.30) | 288 (9.00) |  |
| Marriage status (%) |  |  |  | <0.001^b^ |
| Without spouse | 1108 (16.50) | 489 (13.90) | 619 (19.30) |  |
| Have Spouse | 5620 (83.50) | 3033 (86.10) | 2587 (80.70) |  |
| Smoking (%) |  |  |  | <0.001^b^ |
| No | 5438 (80.80) | 2757 (78.30) | 2681 (83.60) |  |
| Yes | 1290 (19.20) | 765 (21.70) | 525 (16.40) |  |
| Drinking (%) |  |  |  | <0.001^b^ |
| No | 4993 (74.20) | 2532 (71.90) | 2461 (76.80) |  |
| Yes | 1735 (25.80) | 990 (28.10) | 745 (23.20) |  |

Good sleep quality:PSQI<5,;Poor sleep quality:PSQI≥5

Others = other ethnics including Zhuang, Manchu, Hui, Mongolia, Tujia ethnics.

CI: Cognitive impairment; Ref: Reference.

^a^ Pearsons chi-squared test for categorical variables. Significance at p < 0.05.
